# Case Report: Severe COVID-19 Pneumonia in a Patient With Relapsed/Refractory Hodgkin’s Lymphoma

**DOI:** 10.3389/fonc.2021.601709

**Published:** 2021-03-18

**Authors:** Ipek Yonal-Hindilerden, Fehmi Hindilerden, Metban Mastanzade, Tarik Onur Tiryaki, Sevim Tasan-Yenigun, Yusuf Bilen, Selcuk Aksoz, Arif Atahan Cagatay, Meliha Nalcaci

**Affiliations:** ^1^Division of Hematology, Department of Internal Medicine, Istanbul University Istanbul Medical Faculty, Istanbul, Turkey; ^2^Hematology Clinic, Istanbul Bakirkoy Dr. Sadi Konuk Training and Research Hospital, University of Health Sciences, Istanbul, Turkey; ^3^Department of Chest Disease, Adiyaman University Medical Faculty, Adıyaman, Turkey; ^4^Division of Hematology, Department of Internal Medicine, Adiyaman University Medical Faculty, Adıyaman, Turkey; ^5^Department of Infectious Diseases and Clinical Microbiology, Adiyaman University Medical Faculty, Adıyaman, Turkey; ^6^Department of Infectious Diseases and Clinical Microbiology, Istanbul University Istanbul Medical Faculty, Istanbul, Turkey

**Keywords:** coronavirus disease 2019, Hodgkin’s lymphoma, brentuximab, donor lymphocyte infusion, allogeneic stem cell

## Abstract

First identified in China in December 2019, coronavirus disease 2019 (COVID-19) has rapidly evolved into a global pandemic. The presence of haematological malignancies are expected to increase the risk of adverse outcomes from this viral infection due to the immunosuppression brought about by the underlying cancer and the effects of therapy. We present a 55-year-old woman diagnosed with relapsed/refractory Hodgkin’s lymphoma (HL) who had been heavily pretreated with multiagent chemotherapy, autologous hematopoietic stem cell transplantation (autoHCT), allogeneic hematopoietic stem cell transplantation (alloHCT) and was complicated with EBV associated posttransplant lymphoproliferative disease (PTLD) and chronic graft-versus-host-disease (GVHD). The patient was recently treated with brentuximab and donor lymphocyte infusion (DLI) for relapse after alloHCT. She suffered from severe COVID-19 pneumonia and eventually succumbed to acute respiratory distress syndrome (ARDS) and multiorgan failure. Of note, this is the first reported case of COVID-19 in a HL patient who was being treated with brentuximab for relapse after alloHCT.

## Introduction

In December 2019, a novel coronavirus was identified as the cause of a cluster of pneumonia cases in Wuhan, China (1). This novel coronavirus was named as Severe Acute Respiratory Syndrome Coronavirus 2 (SARS-CoV-2). In February 2020, the World Health Organization (WHO) designated the disease coronavirus disease 2019 (COVID-19). Many studies have identified cancer as a comorbidity in COVID-19 patients. The study by Liang et al., which included 1,590 COVID-19 cases, demonstrated that the likelihood of COVID-19 infection was higher in cancer patients compared to the normal population (2). In addition, among cancer patients, the incidence of the need for intensive level care, mechanical ventilation and potential deaths are higher compared to non-cancer patients (2). Immunocompromised patients with hematologic cancers have been historically more susceptible to viral respiratory diseases. That risk is currently more apparent given the increased virulence of the severe SARS-CoV-2 (3, 4). One multicenter study including 105 patients with cancer showed that cancer patients are at higher risk for COVID-19 and encounter a higher frequency of severe events. Moreover, patients with hematologic malignancies such as lymphoma were noted to have relatively higher rates of death, intensive care unit (ICU) admission, and requirement for invasive mechanical ventilation (5). Herein, we describe a patient with Hodgkin’s lymphoma (HL), who suffered from severe COVID-19 pneumonia and eventually died because of acute respiratory distress syndrome (ARDS) and multiorgan failure. To the best of our knowledge, this is the first reported case of COVID-19 in a HL patient who was being treated with brentuximab for relapse after allogeneic hematopoietic stem cell transplantation (alloHCT).

## Case Presentation

**Figure 1:**
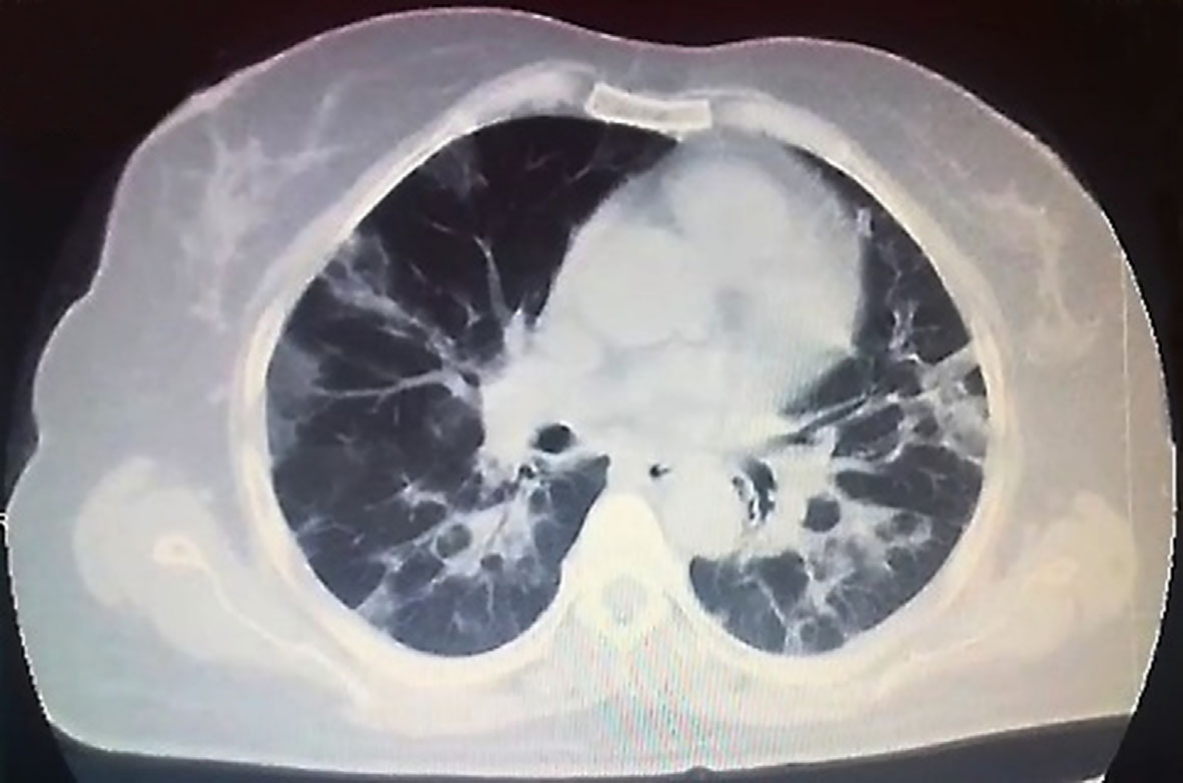
Chest computed tomography (CT) showed patchy peripheral bibasilar ground glass opacities in both lungs.

**Figure 2:**
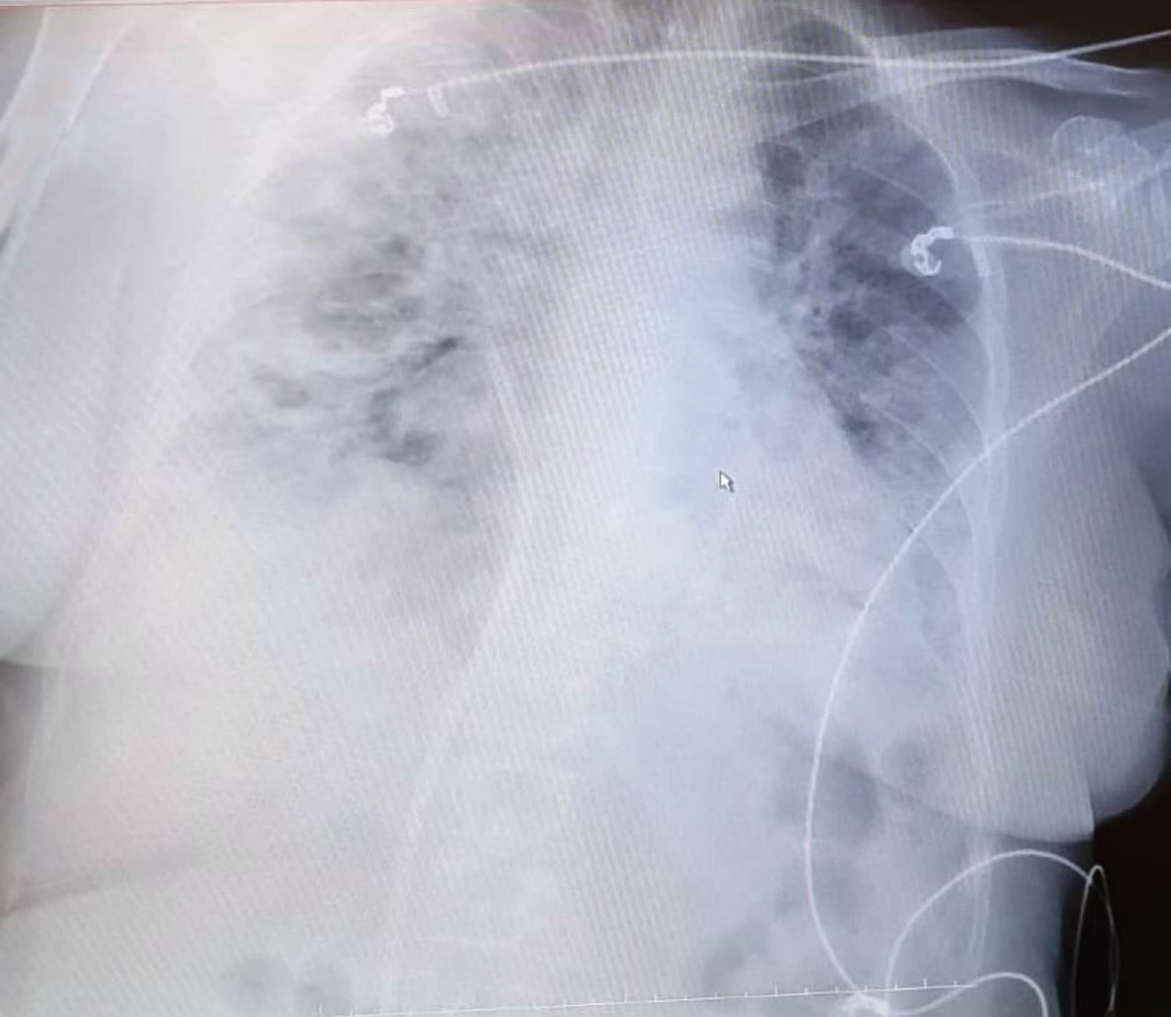
Chest X-ray revealed widespread bilateral alveolo-interstitial infiltrates.

In April 2020, a 55-year-old female with a medical history of classical HL diagnosed on February 2011 was diagnosed with COVID-19. Patient’s treatment history for HL included ABVD (doxorubicin, bleomycin, vinblastine, dacarbazine) at initial diagnosis (February 2011), IVE (ifosfamide, epirubicin, etoposide) as salvage chemotherapy for refractory disease (January 2012) followed by autologous hematopoietic stem cell transplantation (autoHCT) with BEAM (carmustine, etoposide, cytarabine, melphalan) as the conditioning regimen in September 2012. Nivolumab was administered for relapse after autoHCT (April 2016). She developed papillary thyroid cancer in January 2017 and was cured by thyroidectomy and radioactive iodine (RAI) treatment. As salvage chemotherapy, the patient also received brentuximab + bendamustine (February 2017) and GDP (gemcitabine, dexamethasone, cisplatin) (May 2017). Partial remission (PR) was achieved and in March 2018, alloHCT was performed from HLA-matched sibling donor with Fludarabine + melphelan as the conditioning regimen. The source of stem cells was peripheral blood and GVHD prophylaxis included cyclosporine combined with methotrexate. In August 2018, severe chronic graft-versus-host-disease (GVHD) with skin and liver involvement developed. Complete remission (CR) of chronic GVHD was achieved with steroid, cyclosporine, mycophenolate mofetil and methotrexate. In November 2018, she was diagnosed with EBV associated post-transplant lymphoproliferative disorder (EBV associated PTLD) and CR was obtained after eight doses of weekly rituximab. Intravenous immunoglobulin (IVIG) 400 mg/kg/day per 3 weeks was started for acquired hypogammaglobulinemia. In November 2019, the patient developed EBV viremia and was treated with rituximab, valganciclovir and foscarnet. In January 2020, relapse of HL was diagnosed with concurrent appearance of EBV viremia (EBV DNA load 34,694 copies/ml). Brentuximab 1.8 mg/kg and DLI (CD3:5 × 106/kg) every 3 weeks was initiated. He completed three cycles of brentuximab and DLI on March 2020. After three cycles, EBV DNA load decreased to 788 copies/ml and PR was obtained. In April 2020, she presented with shortness of breath and fever. She reported a five-day history of cough. On physical examination, she was pale, her body temperature was 38.3°C and she had hypoxia, with oxygen saturation as low as 88% on room air. Lung auscultation revealed fine bibasilar crackles. Chest computed tomography (CT) showed patchy peripheral bibasilar ground glass opacities in both lungs, findings compatible with severe COVID-19 pneumonia (**Figure 1**). Reverse transcriptase polymerase chain reaction (PCR) assay detected the presence of SARS-CoV-2 RNA in the nasopharyngeal swab. Favipravir, hydroxychloroquine, azithromycin and piperacillin/tazobactam were initiated. IVIG 400 mg/kg was administered. Her complete blood count (CBC) at admission showed the following: Hgb 10 g/dl, Htc 32%, total leukocyte count 4,320/mm^3^, neutrophil 520/mm^3^, lymphocyte 2,940/mm^3^ and platelet count 97,000/mm^3^. Prothrombin and activated partial thromboplastin time were normal. Serum ferritin level was 164 ug/L (23–336). Fibrinogen level was 548 mg/dl (normal range, 200 to 400) and D-Dimer was elevated (1.72 μg/ml; normal range, 0 to 0.5). Low-molecular-weight heparin (LMWH) was started. On biochemical tests, the following were abnormal: lactate dehydrogenase (LDH) 554 U/L (135–248), albumin 2.4 g/dl (3.4–4.5), pro-BNP 2,050 ng/L (70–133). ESR was elevated (48 mm/h; normal range, 2 to 20). C-reactive protein was elevated at 12.6 mg/L (normal range, 0–5) with normal procalcitonin level (0.13 ng/ml; normal range <0.5). During follow-up, she had worsening lymphopenia and her pro-BNP continued to increase. She was transferred to the ICU for respiratory failure and required invasive mechanical ventilation. Her chest X-ray revealed widespread bilateral alveolo-interstitial infiltrates (**Figure 2**). The patient succumbed to severe acute respiratory distress syndrome (ARDS) on the 6th day of admission to the ICU 10 days after initial admission to the hospital.

## Discussion

Centers for Disease Control and Prevention (CDC) reported that immunocompromised patients including those who underwent cancer treatment, bone marrow or organ transplantation, with a history of smoking or prolonged use of corticosteroids have the highest risk for severe COVID-19 (6). In line with the report of CDC, other reports also support that cancer patients are at a higher risk for severe illness from COVID-19, compared to the public (2). Yet, there is no clear recommendation for cancer patients to deal with the COVID-19 epidemic which makes it a challenge for oncology and hematology practice.

Abid et al. summarized recommendations for patients who develop COVID-19 while receiving coventional immunotherapy or immune-engaging therapies (7). They suggested that not critically sick COVID-19 patients should have their conventional immune checkpoint inhibitor (ICI) postponed until complete resolution of infection or wait for at least 2 weeks and immunosuppressive therapy should be considered in addition to immediate cessation of ICI in critically ill COVID-19 patients receiving ICI therapy (7). Similarly, once a patient completely recovers from COVID-19 and remains well for at least 2 weeks after recovery, the patient may be considered eligible to undergo further immune-engaging therapies including CAR T-cell therapy (7). There are few reports of HL cases with COVID-19 infection (8–11). O’Kelly et al. described COVID-19 infection in a refractory HL patient being treated with pembrolizumab (8). Simand et al. reported a HL patient incidentally diagnosed with COVID-19 when reavulated with positron emission tomography–computed tomography (PET/CT) after the fourth cycle of chemotherapy (9). Amin et al. reported an asymptomatic HL diagnosed with COVID-19 infection during routine staging for HL using PET/CT staging (10). Boulvard Chollet et al. reported an asymptomatic COVID-19 infection diagnosed during initial staging of a HL patient (11). To our knowledge, this is the first report of the outcome of a relapsed HL patient after alloHCT, who was being treated brentuximab and subsequently develops COVID-19.

In COVID-19 patients, T-cell responses against the SARS-CoV-2 show good correlation with IgG and IgA antibody titers. This has important implication for long-term immune response (12–14). Total lymphocytes, especially CD8+ T cells are the main inflammatory cells and play an important role in virus clearance (15). Multivariate analysis showed that post-treatment decrease in CD8+ T cells and B cells were reported as independent predictors of poor treatment outcome in COVID-19 patients (15). It has been demostrated that CD8+ T cells tendfonc.2021.601709 to be an independent predictor for COVID-19 severity and treatment efficacy (16). In our patient, lymphopenia deepened during follow-up and the patient did not respond to treatment.

CD30 is expressed in the medullary of the thymus gland on a subset of activated T cells (both CD4+ and CD8+) and B cells (17). A soluble form of CD30 has been found to be elevated in the serum of patients with HL. CD30 blockade compromises the CD4+ T-cell mediated immune system (18). A recent report suggested that brentuximab with/without DLI is an effective salvage therapy for relapse of HL after alloHCT irrespective of pretransplant use of brentuximab (19). We described an HL patient who relapsed after alloHCT and in whom PR was achieved after three cycles of brentuximab and DLI. Cancer and its treatment can compromise the ability of the cancer patients to contain COVID-19 infection. In immunocompromised patients with COVID-19 infection, the likelihood of lethality is higher (20, 21).

Studies support that lymphopenia is a specific risk factor for adverse outcomes from COVID-19 (15). This had led some experts to recommend critical re-evaluation of the need for drugs that inhibit B cells, such as anti-CD20 monoclonal antibodies, during the pandemic (22). There is no clear recommendation about the use of brentuximab during the COVID-19 pandemic. Brentuximab has activity on T-lymphocytes and in a phase I/II study, common adverse events of brentuximab reported included lymphopenia in 80%, neutropenia in 65% and leukopenia in 65% of patients (23). Infections such as pneumonia, bacteremia, and sepsis or septic shock (including fatal outcomes) have been reported in brentuximab treated patients (23). It is recommended to closely monitor patients during brentuximab for bacterial, fungal, or viral infections. In our index case, we think that history offonc.2021.601709 alloHCT, acquired immune deficiency and recent use offonc.2021.601709 brentuximab, led to ARDS and fatal outcome during COVID-19 infection.

Transplant recipients make up a special population of cancer patients, in whom more specialized and tailored attention and care are warranted. There is sufficient concern that COVID-19 could have a significant impact on post-transplant outcomes. A large recent observational cohort study of Center for International Blood and Marrow Transplant Research (CIBMTR) reported a higher risk of mortality among HCT recipients with COVID-19 infection (24). According to the aforementioned study, at 30 days after the diagnosis of COVID-19, overall survival was 68% for recipients of alloHCT and 67% for recipients of autoHCT (24). To date, no ideal marker has been identified to predict the clinical course or outcome of COVID-19 in alloHCT patients. To conclude, our patient is a heavily pretreated patient with relapsed/refractory HL with history of autoHCT, chemo-immunotherapy, alloHCT, EBV associated PTLD, chronic GVHD and immunosuppressive therapy. Abid MA. reported that a sustained immune response occurs in response to viral infections comprising of B-cells, CD4+, and CD8+ T-cells (25). In our index case, aforementioned factors which result in immunosupression and the recent use of brentuximab which causes lymphopenia and compromises the CD4 T-cell mediated immune system resulted in severe disease including pneumonia and ARDS related to COVID-19 infection. The prevention and protection measures to minimize the probability of being exposed to SARS-CoV-2 are very important especially in highly immunosupressive patients. As a whole, cancer is an immunocompromised state, and many cancer treatments can further compromise the immune system. This isfonc.2021.601709 the first report of COVID-19 infection in a HL patient, whofonc.2021.601709 wasfonc.2021.601709 being treated with brentuximab for relapse after alloHCT.

## Data Availability Statement

The original contributions presented in the study are included in the article/supplementary material. Further inquiries can be directed to the corresponding author.

## Ethics Statement

The studies involving human participants were reviewed and approved by Bakirkoy Dr. Sadi Konuk Research Training Hospital. The patients/participants provided their written informed consent to participate in this study. Written informed consent was obtained from the individual(s) for the publication of any potentially identifiable images or data included in this article.

## Author Contributions

All the authors collected data, wrote, and revised the article. All authors contributed to the article and approved the submitted version.

## Conflict of Interest

The authors declare that the research was conducted in the absence of any commercial or financial relationships that could be construed as a potential conflict of interest.
